# FAIR in practice: minimum metadata schema for bioinformatics analytics by machines

**DOI:** 10.1186/s13326-026-00354-9

**Published:** 2026-06-19

**Authors:** Daphne Wijnbergen, Núria Queralt-Rosinach, Valérie Barbié, Emma Verkinderen, Nirupama Benis, Annika Jacobsen, Peter A. C. ’t Hoen, Claudio Carta, Marco Roos, Eleni Mina

**Affiliations:** 1 Leiden University Medical Center, Leiden, The Netherlands; 2 Swiss Institute of Bioinformatics, Geneva, Switzerland; 3 Université Libre de Bruxelles, Brussels, Belgium; 4 Amsterdam UMC Location University of Amsterdam, Amsterdam, The Netherlands; 5 Radboud University Medical Center, Nijmegen, The Netherlands; 6 Istituto Superiore di Sanità, Rome, Italy

**Keywords:** FAIR, Metadata, Machine-actionability, Bioinformatics

## Abstract

**Background:**

One pillar of FAIR principles adoption is Reusability by machines to enable for example more efficient data analytics in fields such as Bioinformatics. However, it is not clear to what extent current metadata exposed by datasets and tools in common repositories enable this. In practice, metadata often lacks in machine actionability due to incomplete standardised metadata and lack of ontological descriptions.

**Results:**

In this work, we identified minimal metadata that is needed to improve the machine actionability of bioinformatics tools and proposed a schema to address current limitations. The schema consists of metadata properties for the identification, selection, validation, and execution of tools. We also aligned this metadata to the metadata of datasets, in order to improve their integration for analytics by machines.

**Conclusions:**

The identified minimal metadata improves the machine actionability of tools and data, and can be incorporated into platforms for tool and data sharing, and in FAIR infrastructures.

**Supplementary Information:**

The online version contains supplementary material available at 10.1186/s13326-026-00354-9.

## Introduction

The reuse of data is crucial to maximize the value gained from existing data resources, which are often the result of significant investments in terms of money and time. For example, in biology, new discoveries can be made using existing datasets by integrating them with other (new or existing) datasets, or by applying new tools and formulating novel hypotheses. To facilitate the reuse of data, the FAIR principles were coined by Wilkinson et al. [1]. These principles show how the findability, accessibility, interoperability and reusability of data can be improved through, for example, metadata provisioning and formal knowledge representation. In practice, the FAIR principles have been applied in various forms to enable human and machine data analysis in heterogeneous data types, including, genomic data [2], (multi-)omics data [3], and rare disease patient registries [4]. The need for adhering to the FAIR principles was especially highlighted during the COVID-19 pandemic, when the reuse of data was hindered by limitations in data sharing (e.g., unstable URLs and limited description of the data) during a crisis in which efficient reuse and integration of data was crucial [5]. In contrast to many data sharing standards, the FAIR principles have been developed with a particular focus on the machine actionability of data for instance by recommending the use of formal knowledge description. Wilkinson et al. define machine actionability as a continuum of amount of information provided about a digital object to a computational agent for identifying the digital objects type, relevance, usability and how to operate on it [1]. Machine actionability is essential for enabling larger scale analyses by allowing computational agents to take over tasks from analysts [1] and will become increasingly important for computational data-driven research especially as AI advances. In the context of this paper, we define the machine actionability of a bioinformatics tool as a combination of tasks that a machine can perform autonomously using the metadata of a tool:

1. A machine can autonomously identify that a tool exists.
2. A machine can autonomously get a quality indication of a tool.
3. A machine can autonomously identify that a tool can operate on a specific dataset (if the dataset is sufficiently described with metadata).
4. A machine can autonomously identify whether a tool can run on a specific system.
5. A machine can autonomously obtain and execute a tool and retrieve its results.

In order to scalably reuse data in practice, it becomes a necessity to perform data analysis on a FAIR ecosystem so that datasets from heterogeneous sources can be found simultaneously without consulting every source individually. This is especially important for rare diseases for which data is scarce. However, being able to link datasets from different sources into a FAIR knowledge graph (A knowledge graph [6] following FAIR principles, primarily principles I1 and I2, which are formal language representation e.g. through using RDF, and use of vocabularies that follow the fair principles) is a huge challenge. In the Swiss Personalized Health Network (SPHN) for example, a national FAIR data schema for health data (https://www.biomedit.ch/rdf/sphn-schema/sphn) and a corresponding toolstack is developed to ensure hospitals deliver interoperable data that can be easily used in research projects [7]. Similarly, in the European Joint Programme on Rare Diseases (EJP RD), a FAIR infrastructure was developed in order to allow the discovery and privacy preserving accessibility of various resources such as patient registries, biobanks and datasets in an interoperable manner within its Virtual Platform [8, 9]. Another example currently undergoing development is the FAIR Data Train [10]. This is a FAIR federated ecosystem consisting most importantly of FAIR Data Stations containing datasets and FAIR Data Trains containing algorithms, which both require metadata to empower computational agents to perform analysis.

Despite the introduction of the FAIR principles, current metadata standards and practices in data repositories, as for example in omics data repositories, remain a bottleneck for analytics by humans, and even more so for machines [11–13]. One reason for this bottleneck is that current metadata standards and practices are often incomplete, and thus are missing elements that would greatly benefit the reuse not only of datasets but also of computational tools in bioinformatics. Additionally, most metadata are focused on human readability, and as a result are not accessible for machines. For example, a lot of information only exists in textual descriptions of data instead of using ontological definitions.

Like datasets, tools in bioinformatics can also benefit from reuse. For this reason, the FAIR principles are increasingly being applied to software. For example, in previous work, the FAIR4RS initiative has created an adaptation of the FAIR principles for analytical tools and pipelines to improve their reproducibility and reusability [14, 15]. In this adaptation the principles have been modified to be applicable to software. De Visser et al. have followed up on the FAIR4RS initiative by recommending practical applications to increase the FAIRness of workflows [16]. For example, uploading a workflow in Common Workflow Language [17] to WorkflubHub [18] with sufficient metadata will make it more reusable [19, 20]. Although the FAIR4RS principles mention the need for rich metadata, it does not aim to set specific guidelines on what metadata is needed to achieve enough richness for machine actionable analysis. In related work, efforts have also been made to increase the interoperability of tool metadata in different repositories by mapping the properties of different repositories to each other with the Crosswalk mappings [21] created by the CodeMeta Project [22]. Despite these efforts, much software is still contained in repositories with insufficient metadata for machine actionable analysis and hampered machine readability of the metadata. For example, memory requirements are not reported in repositories such as bio.tools [23], CRAN [24] and BioConductor [25] though this is essential for understanding whether a system can run the tool. Similarly, software maturity is not reported in Schema.org, CRAN and BioConductor, despite its usefulness in finding tools that are established and thus likely to be reliable. Our work aims to complement current metadata by proposing minimal metadata properties that enable machine actionable analysis, while aligning with existing schemas.

Despite much focus on the FAIRness and metadata of data, and to a lesser degree on tools, currently popular metadata standards for FAIR data and tools have not been investigated in a connected manner. This connected view is important, since for machine based FAIR analytics, it is necessary that both tools and data are FAIR. The metadata should ideally also be aligned to facilitate integrated use of data and tools. In this paper, we propose a minimal metadata schema that addresses limitations of current metadata for tools and data and enables machine actionability of computational bioinformatics tools and data by connecting their metadata. To achieve this goal, we review current metadata standards and identify what metadata for tools is minimally necessary for the purpose of machine actionability. We also identify and propose which metadata is needed for interactions between tools and data to enable FAIR based data analytics.

## Methods

### Requirements and strategy

To steer our minimal metadata schemas (one for the machine actionability of tools and one for the alignment of this schema to the metadata of datasets) towards a clearly established direction, we defined four goals:

- The metadata should facilitate the reuse of data and tools.
- The metadata should facilitate the connection between data and tools.
- The metadata should follow the FAIR principles and consequently be usable by machines.
- The metadata should be valuable for the biomedical science and bioinformatics domains.

Our strategy followed a community-driven approach for decision-making that consisted of forming a group of stakeholders that are involved in the ELIXIR Rare Disease Community. These stakeholders represent various domains within bioinformatics and healthcare data such as genomics, transcriptomics, health infrastructure and the FAIR principles. Within this group, we organized regular calls in order to jointly investigate, identify and then decide what the minimal metadata schemas should consist of, starting from selected tools widely used in the RD community.

### Approach

**Fig. 1:**
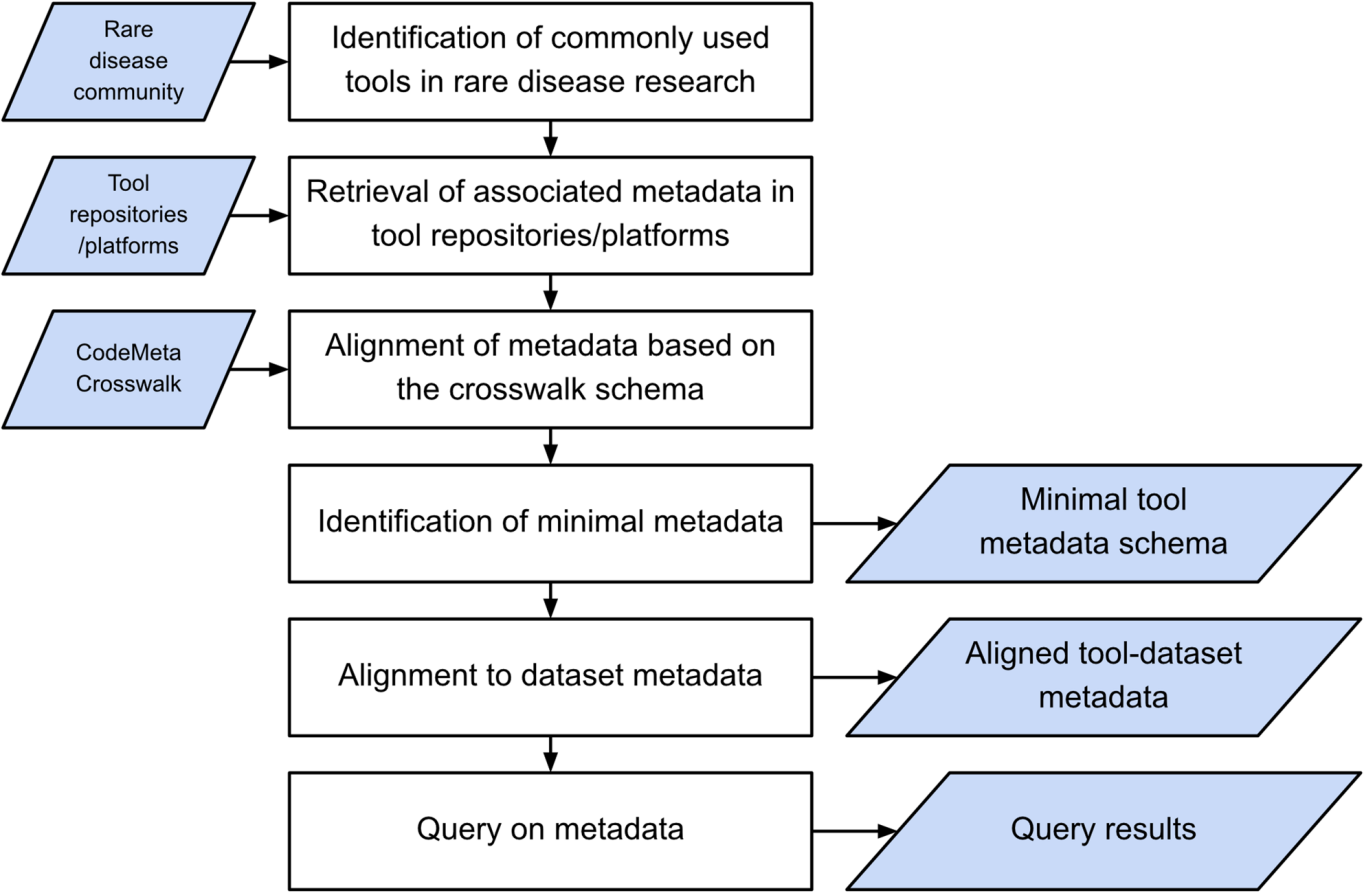
Workflow for the identification of the minimal tool metadata, and alignment to dataset metadata. Sources of data and knowledge are shown on the left side of the figure, while the results are displayed on the right side of the figure. The processes by which the results were produced from the inputs are shown in the middle

Our approach consists of several steps (Fig. 1), which are described in more detail in the following sections. First, we selected a list of tools that are used in the Rare Disease Community by conducting a survey and querying bio.tools for tools tagged with “Rare Disease”. For a selection of popular tools from this list, we identified metadata that is available for these tools in common repositories and platforms for tools. We then aligned these lists to each other to compare them and to identify which metadata is necessary for machine actionability as defined in the scope of this paper. Finally, we made an alignment between metadata for data, and metadata for tools to increase their interoperability. On the resulting minimal metadata, we executed several queries to compare machine actionability of resources with and without FAIRification.

### Identification of minimal metadata for tools

To gain insight into the current state of metadata for bioinformatics tools and to build upon this, we identified which metadata is currently published in repositories. For this purpose, we selected a sample of bioinformatics tools used in the rare disease community. We defined three inclusion criteria for these tools: the tool must be a bioinformatics tool, it must be used in rare diseases, and it must have metadata in one or more registries. We sent out an email survey to members of the ELIXIR Rare Diseases Community [26] and the EJP RD [27], asking which tools they used in their research and the function of each tool. Additionally, we identified tools used in rare disease research by querying bio.tools for tools tagged with “Rare Disease” using the following API query: “bio.tools/api/tool/?collectionID=%22Rare+Disease%22&format=json&page = 1”. We programmatically obtained each page of the results to create a list of tools. It is important to note that the results of this query will change over time as the content of the bio.tools database evolves. We made a selection from these lists to limit the number of tools to a maximum of two per contributor. We prioritized tools based on our familiarity with them in order to ensure our correct understanding of their metadata. This selection contained the following tools: LIMMA [28], ORVAL [29], orsum [30], Variomes [31], RD-Connect GPAP [32], MOGAMUN [33], BridgeDB [34], VEP [35], PathVisio [36], mixomics [37, 38], ODAMnet [39], Robust Rank Aggregation [40] and DESeq2 [41]. For these tools, we obtained the available metadata in one or more platforms that facilitate the sharing of tools, specifically bio.tools [23], CRAN.R [24], Bioconductor [25] and the EJP RD Virtual Platform [8, 9]. Metadata was retrieved manually using the available web interfaces and APIs during a time window from October 9 to October 27 in 2023.

In order to further investigate the retrieved metadata, we created a spreadsheet with the metadata properties on the rows, and different tools and repositories on the columns. For each combination of property and tool/repository, we filled in the metadata property or metadata value. In order to initiate the alignment of metadata properties for different software repositories, we used Crosswalk metadata mappings [21] from the CodeMeta Project [22] as a base since this project is devoted to providing a table to connect the diverse metadata. Metadata not included in the Crosswalk mappings was added at the bottom. Next, using the spreadsheet, based on each platform and tools metadata, we identified for each property whether it is necessary for analytics by machines with expert consensus through majority vote. Additionally, we identified which FAIR principle is satisfied by each metadata property in the set of minimal metadata. In order to assign FAIR principles to each property, they were discussed in meetings with several FAIR experts. Here the function of the properties were compared to the definitions of the principles in order to form an informal expert consensus for each property.

### Alignment of metadata for data and tools

To create an alignment of metadata between data and tools, we investigated the minimal tool metadata that we identified previously, and formed an expert consensus on whether this property is also applicable to data, and can be used by a computational agent to see if a specific tool can be used with a specific dataset (e.g. by matching data types and input types), or be used to combine the two properties into a combined property (e.g. by adding the storage requirements of a tool and dataset to get total storage requirements). Expert consensus was then formed through majority vote with the same group of experts as the selection of minimal metadata in order to determine whether the properties met one of these criteria and should thereby be included in the aligned metadata.

### Evaluation of using metadata with and without FAIRification

**Fig. 2:**
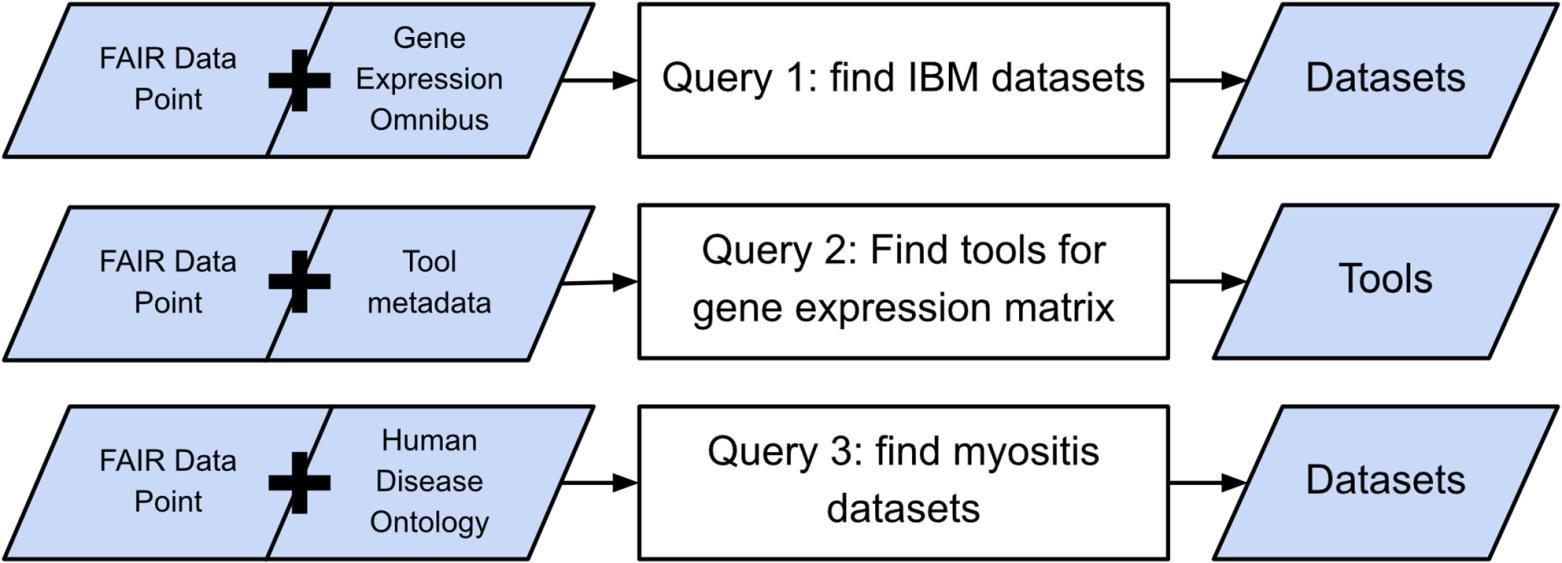
Workflows for three queries that we executed to investigate the impact of FAIRifications on finding tools and datasets. Query 1 finds IBM datasets in FAIR Data Points and the Gene Expression Omnibus. Query 2 combines FAIR Data Points and tool metadata to find tools that are applicable to a specific dataset. Query 3 combines FAIR Data Points and the Human Disease Ontology to find datasets belonging to several myositis diseases

We compared the performance of FAIR metadata to non-FAIR metadata to assess their machine actionability. In order to do so, we created three different queries (overview shown in Fig. 2).

In the first query, we searched for datasets about the disease “Inclusion Body Myositis” in both the Gene Expression Omnibus (GEO), and in FAIR Data Points [42] in December 2023. Here the FAIR Data Point was designated as a more FAIRified resource due to adhering to FAIR principles I1 and I2 which are “(meta)data use a formal, accessible, shared, and broadly applicable language for knowledge representation.” and “(meta)data use vocabularies that follow FAIR principles” respectively [1]. Note that both resources are FAIR to some degree as they both adhere to several other FAIR principles. In GEO (https://www.ncbi.nlm.nih.gov/geo/) we used the search term “Inclusion Body Myositis”, while in the FAIR Data Points (https://index.vp.ejprarediseases.org/), we searched for datasets with an ontology term for “Inclusion Body Myositis” (DOID:3429 from the Human Disease Ontology [43]). We calculated the number of true and false positives from the results that we retrieved by querying both repositories.

In the second query, we performed a query that matches datasets to tools by using ontology terms from a shared ontology. Specifically, we used terms from the EDAM ontology [44], which is an ontology for bioscientific data analysis and data management, and includes concepts such as data types, formats and operations. In this query, we queried an Inclusion Body Myositis dataset for its ontological description by DCAT themes [45]. We then searched for tools that have one of these themes as an input.

Finally, in the third query, to investigate the use of information embedded in ontologies, we performed a query that finds datasets for a group of related disorders by using the semantic information that is embedded in the Human Disease Ontology [43]. Specifically, we searched for datasets for all diseases in the group of “myositis” (DOID:633). All queries are available at https://github.com/LUMC-BioSemantics/Tool-and-data-queries.

## Results

### Recommended minimal metadata of tools for machine actionability

**Fig. 3:**
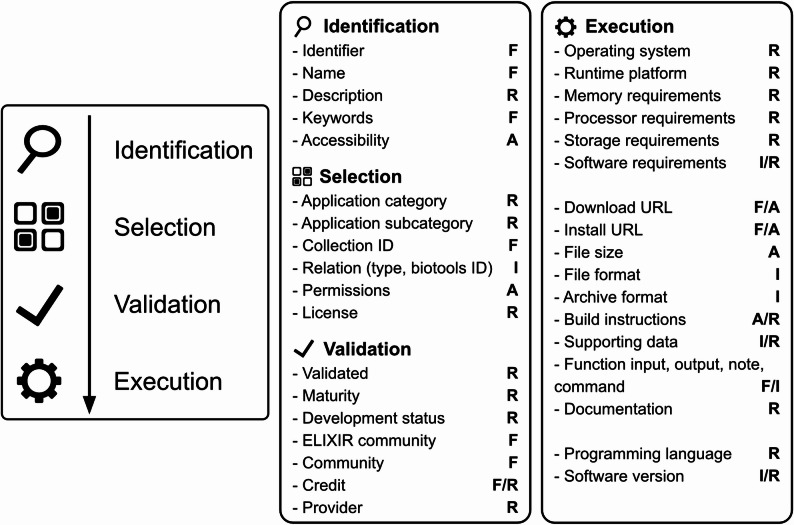
The identified minimal metadata for tools divided in four sequential categories: identification, selection, validation and execution. Descriptions of each property are given in Additional file 1

We identified 32 tools used in rare disease research in the survey, and 248 tools tagged with rare disease in bio.tools (out of 28,368 total tools). For 13 tools that were selected based on their popularity, we identified their metadata in several platforms. Based on this metadata, a selection of minimal metadata for machine actionable analysis was created, as shown in Fig. 3. Since the resulting metadata list was relatively long, we divided the metadata into four groups representing four subsequent steps in machine actionable analysis. First, to be identified, a tool needs an identifier and a name. Second, to select tools that are applicable for a dataset, metadata such as the application category, permissions and license are needed. Third, for the validation of the tool, multiple quality indicators, such as the maturity, development status, and use in different communities are valuable. Finally, for the execution of the tools, metadata covering some computational aspects are necessary. This includes metadata such as hardware and software requirements, but also metadata that is needed to know how the tool should be executed. Each property is described in more detail in additional file 1. Additionally, an alignment of the metadata properties to the source repositories is shown in Table 1 and Additional file 1, and can be used to see where each property fits in the source metadata model. For each metadata property, we also indicate the relevant FAIR principles.

**Table 1:** Snapshot of mappings for the identified minimal metadata in several repositories. The full table is shown in Additional file 1

Property	CodeMeta/Schema.org	Bio.tools	EJP RD/DCAT	CRAN	Bioconductor
Identifier	schema: Thing/identifier	tool::biotoolsID	dct: identifier	Package	-
Name	schema: Thing/name	tool::name	dct: title	Title	Title
Description	schema: Thing/description	tool::description	dct: description	Description	Description
Keywords	schema: CreativeWork/keywords	tool::topic (term, uri - both EDAM)	dct: keyword	-	-
Accessibility	-	tool::accessibility	dct: accessRights	-	-

### Recommended common metadata to connect tools and datasets

**Fig. 4:**
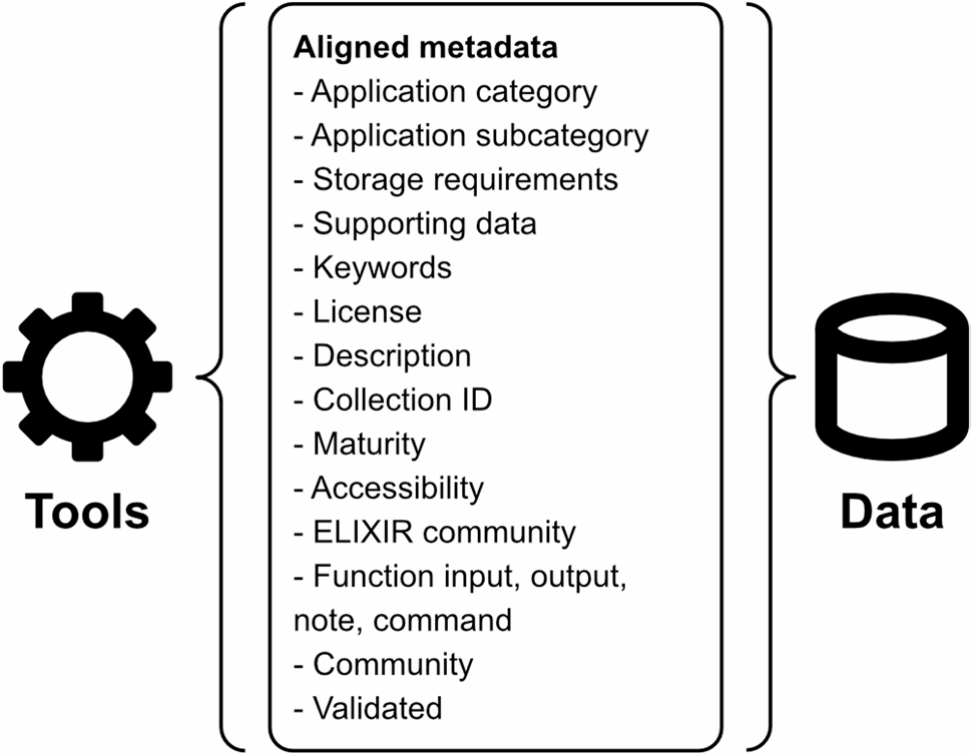
Aligned metadata for both tools and data. These metadata properties can be matched between tools and data, and thereby allow interaction between tools and data to perform bioinformatics analysis

An overview of the metadata properties that we have aligned between datasets and tools is shown in Fig. 4. This alignment resulted in a schema that contains metadata that is relevant for both datasets and tools, and can be used to establish connections between the two for data analysis. One important property for alignment is the data input and output type, which can be described for example with EDAM ontology terms. Here, a tool might be described as requiring FASTQ files (http://edamontology.org/format_1930) as input, while a dataset can be described as being in the FASTQ format. These metadata properties can then be used to match the tool to the dataset. Another example is the license, since it is necessary to have the rights to use both the data and the tool, when performing an analysis. For example, a computational agent may check the licenses for both a dataset and a tool to understand under what conditions they can be accessed, used and shared by the organisation. Finally, the storage requirement for both data and tools need to be added together to get the total amount of storage that is required to perform an analysis.

### Machine actionability of tools and data with and without FAIRification

We performed queries (https://github.com/LUMC-BioSemantics/Tool-and-data-queries) for three tool and data identification scenarios. When querying GEO for the term “Inclusion Body Myositis”, we found 11 true positives and 4 false positives. The false positives were retrieved because the name “Inclusion Body Myositis” or “IBM” is mentioned in the description as free text either because the study is related to Inclusion Body Myositis, or because the statistical software SPSS from the company “IBM” has been used in the study. In contrast, when querying FAIR Data Points for the ontology term “Inclusion Body Myositis” (DOID:3429), we found 4 true positives and 0 false positives (in this case, there are less results since adoption of the FDP is lower). In addition, findability time for GEO was 5 min and 9 s due to the manual curation needed, while for FAIR metadata this was 13 s.

For the second query, we queried one of the identified transcriptomics datasets for Inclusion Body Myositis in a FAIR Data Point Index for its data format. Here we found the “expression data” (EDAM:2603) EDAM term. Using this EDAM term, we then queried FAIR Data Points for tools that have this EDAM term as their input. As a result, out of 4 tools that were indexed in the FAIR Data Point Index, we found the DESEQ2 tool, which performs differential gene expression testing, and is applicable on this dataset.

In the third query, we first queried the ORDO ontology [46] for diseases that are a subclass of “myositis”. Using this list of diseases, we subsequently queried FAIR Data Points for datasets that are described with at least one of these disease terms. We found 4 datasets for Inclusion Body Myositis out of the 21 total dataset that were connected to this FAIR Data Point index.

## Discussion

In this work, we identified important gaps in current metadata standards for machine actionable bioinformatics analysis. Our main findings are a lack of machine readability, and a limited alignment of metadata for tools and datasets. As a solution, we propose minimal metadata for tools, and an alignment of metadata for tools and datasets.

We identified a minimal set of metadata properties that we propose to be minimally necessary for machine actionable analysis. By drawing from existing repositories, we include properties that were only included in one of some of these repositories. This includes memory requirements and software maturity, which are important for machine actionability, as it allows the machines to avoid runtime failures due to a lack of allocated memory and indicates if the software is reliable enough for use. Additionally, we aligned the tool metadata to dataset metadata to increase their interoperability. For example, in our second query, describing inputs and outputs of tools, and format of data, allowed a computer agent to know which tool works for which data. A real world example for this alignment would be machine actionable variant effect prediction on vcf data using VEP (Variant Effect Predictor) within a DRE (digital research environment). In this case, the machine would need to reserve storage space in the DRE. This is only possible to do if the storage requirements of both the tool and data is known, as datasets can be large, and tools such as VEP can rely on large plugins. In this case, the machine would identify the storage requirements of both the genetic variation data and the VEP tool, sum these values together, and then request sufficient storage space to perform the analysis. The identified tool metadata and its alignment to dataset metadata meets the four goals that we defined. They facilitate the reuse of tools and data, facilitate the interoperability of tools and data, follow the FAIR principles where possible, and are relevant for the biomedical science and bioinformatics domains.

Queries on our metadata schemas showed that the machine actionability aspect of the FAIR principles has several important benefits in research. For example, we were able to find datasets with more precision by incorporating ontology terms into the search instead of plain text. This highlights the importance of the use of ontologies to avoid ambiguity for terms and acronyms that have multiple meanings. The second query showed that ontologies also improved interoperability between datasets and tools, by allowing them to be described with identical or mapped ontology terms, ensuring the machine understands that the resources annotated with these terms have the same meaning without being affected by differences in spelling or ambiguity of natural language. Finally, we found that the application of FAIR principles including the use of ontologies enabled machines to use knowledge embedded in ontologies. A representative example was to obtain diseases that belong to a certain disease group, when querying for a particular disease. This makes queries more powerful, and thus improves machine actionability.

There are several limitations to note in this work. The scope of our work is focused on biomedical sciences and bioinformatics and although our work could be applicable to other domains, existing data sharing models and standards differ between fields. So while the properties itself can be reused, it would be necessary to make adjustments, such as using a domain specific ontology instead of the EDAM ontology for the function property. Similarly, the categorization of tools would need to be adapted for different domains. Further, the comparison between analysis with and without FAIRification has limited quantification, which limits the strength of its conclusions. With a higher adoption of the FAIR principles comparable to platforms that are not FAIRified, a more comprehensive comparison could have been made. Finally, some information is still missing that would be needed to achieve fully automatic analysis, especially installation and execution of tools. As a consequence, the installation of a tool will require some standardization, such as installation through Docker or a package manager, and execution of a tool would require similar standardization, for example through a workflow language such as CWL. Setting optimal parameters for these workflows would also remain a challenge (e.g. choosing which variables to include in a linear model) since it requires good understanding of both the data, and the impact of the parameters of the tool.

In future work, the minimal metadata that we identified can serve as a reference for data and software sharing platforms to improve the machine actionability of their metadata and improve the alignment between tools and datasets. This would be facilitated by the provided alignment to existing schemas in additional file 1. Existing schemas for tool metadata could be extended with new properties to increase machine actionability while avoiding further fragmentation of metadata schemas. This would have the benefit of allowing existing workflows to be used and extended. Our minimal metadata can also be implemented in FAIR ecosystems such as the SPHN and EJPRD, and be promoted within ELIXIR. The minimal metadata would especially benefit federated infrastructure such as the FAIR Data Train [10], which requires a high degree of machine actionable communication between FAIR Data Stations (containing datasets) and FAIR Data Trains (containing tools). Our minimal metadata schema can serve as a starting point in implementing this communication, for example, by describing the memory requirements of a train, so that sufficient memory is allocated by a FAIR Data Station when running a tool. The minimal metadata that we identified can also be expanded further in order to achieve an increasingly higher degree of machine actionability for tools and datasets. Efforts could for example focus on improving the interaction between tools and datasets by enabling more granular file format descriptions of datasets and of tool inputs and outputs thereby connecting the two in a way that is precise enough for computer agents to work increasingly more independently. Further work should also be done on creating a pilot study on creating an automatic workflow using the proposed minimal metadata to find, install and use a tool in a real life scenario and find potential bottlenecks in achieving this. In addition, a benchmark framework to measure the machine actionability of a tools metadata would also be valuable for assessing different metadata schemas for tools.

## Conclusion

In this work, we propose two metadata schemas to address current limitations of metadata. One is focused on minimal metadata for tools, and the second one is focused on alignment between metadata of data and tools. Through queries, we showed that our metadata schema performs well in terms of precision, aligning data with tools, and allowing for more complex and powerful queries. These schemas can be used in platforms for bioinformatics tools and in FAIR infrastructures, but can also apply beyond the Rare Disease domain, and serve other areas.

## Supplementary Information

Below is the link to the electronic supplementary material.

Supplementary Material 1

## Data Availability

The queries that support our findings are available on GitHub, [https://github.com/LUMC-BioSemantics/Tool-and-data-queries].
